# *Cryptosporidium* species subtypes and associated clinical manifestations in Indian patients 

**Published:** 2017

**Authors:** Shehla Khalil, Bijay Ranjan Mirdha, Ashutosh Panda, Yogita Singh, Govind Makharia, Jaishree Paul

**Affiliations:** 1 *Department of Microbiology, All India Institute of Medical Sciences, New Delhi, India*; 2 *Department of Gastroenterology and Human Nutrition, All India Institute of Medical Sciences, New Delhi, India*; 3 *School of Life Sciences, Jawaharlal Nehru University, New Delhi, India*

**Keywords:** *Cryptosporidium*, Genetic variations, Glycoprotein gp60, phylogeny

## Abstract

**Aim:**

Present hospital based study was carried out at our tertiary care centre with an aim to study the distribution of *Cryptosporidium* species subtypes in patients with complaints of diarrhea.

**Background:**

*Cryptosporidium* species are one of the important causative agents of parasitic diarrhea, amongst which *Cryptosporidium hominis *(*C.hominis*) and *Cryptosporidium parvum *(*C.parvum) *are the two major species that are associated with human cryptosporidiosis.

**Methods:**

Four hundred and fifty (n=450) diarrheic patients complaining of different types of diarrhea were enrolled in the present study. Both microscopic and molecular diagnostic methods were used for the detection as well as for identification of *Cryptosporidium *species and its speciation and subtyping.

**Results:**

Forty one (n**=**41) and forty three (n=43) patients were positive for *Cryptosporidium* species by microscopy and Polymerase chain reaction (PCR) assay respectively. Of these 43 cases, 70% (30/43) were identified as *C. hominis* and 21% (9/43) was as* C. parvum, *7% (3/43) was as *Cryptosporidium felis (C.felis)* and 2% (1/43) as *Cryptopsoridium viatorum *(*C. viatorum*) respectively *.* Upon subtyping of C*. hominis *and *C. parvum*, 16 subtypes belonging to 8 different subtype families could be identified. The frequency of different families were Ia (13%, 5/39), Ib (15%, 6/39), Id (18%, 7/39), Ie (30%, 12/39) and IIa (5%, 2/39), IIc (8%, 3/39), IId (8%, 3/39) and IIe (3%, 1/39).

**Conclusion:**

Our study results strongly suggest and reinforces the fact that most of the human cryptosporidiosis is anthroponotic and we expect that present molecular epidemiological data will provide more insight to unravel the changing clinical paradigm of human cryptosporidiosis at large.

## Introduction

 In recent times, *Cryptosporidium *species are one of the most important causative agents of diarrhea in both immunocompetent and immunocompromised individuals. Humans can acquire infections either by direct contact with infected persons (anthroponotic transmission) or from animals (zoonotic transmission) or by ingestion of contaminated food or water (1,2). Human cryptosporidiosis is primarily caused by *Cryptosporidium hominis* (*C. hominis*) and *Cryptosporidium parvum* (*C. parvum*); however other species such as *C. meleagridis*, *C. felis* and *C. canis* have also been reported to cause human cryptosporidiosis but with much lower frequency (3). 

 It has been observed that the distribution of *Cryptosporidium* spp. in humans varies to some extent with geographic locations and socioeconomic conditions of a particular country. *C. hominis *is the most common species that infect humans in most of the developing and developed nations (3-6) except Middle Eastern countries, where *C. parvum *is the most common infecting species (7). Apart from above variables infecting *Cryptosporidium* spp. does vary with age of the infected individuals (8, 9). *C. hominis* has been commonly reported in children compared to *C. parvum* albeit some studies that defy such association (10, 11).

 Human cryptosporidiosis in immunocompetent individuals may present with three major clinical presentations such as i) asymptomatic carriage, ii) acute diarrhea and with iii) persistent or chronic diarrhea. The incubation period may vary from three to fourteen days and the illness is usually self-limiting (12). However, Cryptosporidiosis tends to be much more severe in immune-compromised individuals with impaired or defective cell-mediated or humoral immunity or both (13, 14). 

 In recent years various studies have delineated the causal association of different clinical manifestations of cryptosporidiosis with that of infecting *Cryptosporidium *species, subtype family or subtypes (11,12, 15-17). The present hospital based cross sectional study was conducted to detect and subtype *Cryptosporidium* species from different group of patients attending our tertiary care centre with complaints of diarrhea. 

## Methods

**Ethical approval**

Ethical approval for the present study was obtained by institutional ethical committee of All India Institute of Medical Sciences, New Delhi, India. All the participants were apprised about the study protocol. Informed consent was obtained from all adult patients as well as and from guardians/ Parents of Children enrolled in the study. During the meetings, enrolled individuals were informed that their participation was voluntarily and they have all the rights to withdraw from the study at any point of time without giving any reason, however withdrawal from the study did not affect the treatment and medical care provided to these patients. Patients’ personal details were kept confidential.

**Study groups/Patients**

The present study was conducted in the Department of Microbiology. Study population were enrolled from both in-patient and out-patient departments of Gastroenterology and Human Nutrition, Paediatrics, Internal Medicine and Dr. B. R. Ambedkar Institute of Rotary Cancer Hospital (IRCH) of our tertiary care referral health centre. The patients who had received any luminal anti- parasitic treatment in last three weeks were excluded from the study.

**Clinical specimens**

Three (n=3) consecutive stool samples on three consecutive days were obtained from four hundred and fifty (n=450) patients with diarrhea (cases) and two hundred (n=200) individuals without diarrhea (controls). Diarrhea was defined as the passage of liquid or unformed stool at an increased frequency and was further categorized as ‘acute’ if the diarrhea was for a period of less than two weeks and ‘persistent’ if it was present for two to four weeks. ‘Chronic’ diarrhea was defined when the complaints were for more than four weeks’ duration (http://www.who.int/topics/diarrhoea/en/) (WHO, 2009). All (n=450) cases were comprised of both clinically apparent immunocompetent and immunocompromised patients that included HIV sero-positives, transplant recipients, patients with malignant diseases or other immunodeficiency disorders. Controls enrolled in the study included healthy individuals without having any gastrointestinal complaints. Three consecutive samples obtained from each patient during the study were considered as ‘one’ for the convenience of subsequent analyses. 

**Clinical** **manifestations**

Information regarding clinical manifestations was recorded by interviewing the patients as well as from caregivers in case of paediatric population. Data included relevant gastrointestinal symptoms such as abdominal pain, fever, general malaise, nausea, vomiting, frequency and duration of diarrhea and presence of blood or mucus in the stools. Duration of diarrhea, vomiting, fever, abdominal pain and weight loss were included in the study for subsequent analysis. 

**Statistical** **analysis**

Statistical analysis was performed using STATA 12.2 software and wherever applicable *p*-values were calculated. The discriminatory power of gp60 gene was calculated online using http://insilico.ehu.es/mini_tools/discriminatory_power/.

**Microscopic examination**

Processing of fecal samples was carried out depending upon the consistency. In case of watery, samples direct wet-mounts (both in normal saline and 3% Lugol’s iodine) and fecal smears were prepared, whereas for the formed stools, additional standard formol-ether concentration technique was used. Wet mounts as well as fecal smears were prepared from the sediments for examination (18). Fecal smears were subjected to Modified Ziehl-Neelsen (mZN) staining (19).

***Cryptosporidium*** **spp. detection, identification and subtyping by molecular methods**

*Extraction of genomic DNA*

Immediately after collection, all the clinical samples (n=650; ie. 450 cases and 200 controls) were subjected to DNA extraction using commercially available QIaAmp stool minikit (Qiagen, Germany) as per the manufacturer’s instructions, with some modifications. The modifications included (i) mechanical disruption of oocysts using glass beads, (ii) incubation of clinical sample at 95ºC for 60 minutes after addition of lysis buffer, (iii) incubation at 70ºC for 30 minutes after addition of Proteinase K and (iv) DNA elution was done in 50µl of elution buffer instead of 200µl recommended in the protocol. The extracted DNA was stored at -80°C till further analysis.

SSU-rRNA PCR-RFLP assay

PCR assay targeting small subunit ribosomal RNA (*SSU rRNA)* gene of *Cryptosporidium *spp. was performed a using nested protocol. The external round of PCR assay was performed using SSU-F1 (5’- TCTAGAGCTAATACATGCG-3’) as the forward primer and SSU-R1 (5’-CCCATTTCCTTCGAAACAGGA-3’) as the reverse primer, whereas, SSU-F2 (5’-GGAAGGGTTGTATTTATTAGATAAAG-3’) and SSU-R2 (5’-CTCATAAGG TGCTGAAGGAGTA-3’) were used as the forward and reverse primer for the nested round respectively (20). The reaction mixtures for both external and internal rounds of PCR assay contained 2.5µl of 10X buffer, 3µl of 25mM MgCl_2_, 2µl of 10mM dNTPs, 0.5µl of 20pm of each primers and 0.8µl of 3 units of *Taq*DNA polymerase.PCR assays were performed by an initial denaturation at 95ºC for 10 minutes followed by 35 cycles of denaturation at 94ºC for 10 seconds. Annealing was carried out at 61ºC (57ºC for internal round) for 10 seconds, followed by extension at 72ºC for 15 sec and final extension at 72ºC for 10 minutes. The assays were completed with a final hold step at 4ºC in C1000^TM^ Bio-Rad thermocycler (Bio-Rad, USA). The intensity and size of all amplicons were assessed by electrophoresis in 1.5% ethidium bromide stained agarose gels using Tris Borate EDTA (65 mMTris-HCl, 27 mM boric acid, 1 mM Ethylene diamide tetra acetate, pH 9; Bio-Rad, USA) as the buffer and 100bp molecular marker (Fermentas).

PCR-RFLP assay was then performed using *Ssp*I and *Ase*I (New England Biolabs, USA) restriction enzymes for genotyping of *Cryptosporidium* spp. Six microlitre (6µl) of PCR product was digested with 6 units of each enzyme in separate reactions at 37ºC for a period of 4 hours. 

*Subtyping using gp60 gene*

DNA samples from all PCR-confirmed *C. hominis *and *C. parvum *cases were subjected to further amplification of a 850-bp fragment of the *Cryptosporidium * specific *gp60* gene using F1 5’-ATAGTCTCCGCTGTATTC-3’and R1 5’-GGAAGGAACGATGTATCT-3’ as external primers and F2 5’-TCCGCTGTATTCTCAGCC-3’ and R2 5’ GCAGAGGAACCAGCATC-3’ as internal primers (21). Annealing was carried out at 50ºC for 60 seconds. 

*Sequencing*

The positive amplified product obtained using both SSU-rRNA and gp60 gene PCR assay was excised from agarose gel and purified using QIAquick gel extraction kit (Qiagen, Valencia, USA) according to the manufacturer’s instructions. The PCR fragments after purification were further subjected to sequencing using an automated DNA sequencer (ABI Prism 310) using BigDye Terminator Chemistry. DNA chromatograms were examined using BioEdit software versions 7.1.3. Both forward and reverse sequences were pair-wise aligned along with the reference sequences using *Clustal W* software and were manually refined to obtain a better consensus sequence. 

*Phylogenetic* *analysis*

To further support the results of subtyping, two neighbour-joining trees were built using Kimura two-parameter model in MEGA 6, each for *C. hominis* and *C. parvum*. The reliability of all phylogenetic groupings was determined through a bootstrap resampling analysis (1,000 replicates). Reference sequences of both* C. hominis and C. parvum* subtypes identified from Indian studies form Kolkata (22) and Chandigarh (23) were included from the NCBI GenBank database as controls for the alignment. 

*Nucleotide sequence Accession numbers*

Representative sequences of the *Cryptosporidium* spp. at SSU-rRNA and gp60 genes in this survey were submitted to the GenBank under accession numbers KX056082-KX056097, KX174306-KX174309 and KU169226 -KU169235. 

## Results

**Study group**

Of the total four hundred and fifty (n=450) cases enrolled, three hundred and seven (n=307) were adults and one hundred and forty three (n=143) were children with a mean age of 35.75±13.4 years and 5.28±3.44 years, respectively. A total of two hundred age-matched healthy individuals comprising of 100 adults (males 55, females 45) and 100 children (males 57, females 43) without any gastro-intestinal disorders were enrolled as controls. The mean age of the adults and children was 29.7±12.5 and 5.7±3.8 years, respectively. Distribution of these patients along with their underlying condition has been shown in Table 1.

***Cryptosporidium*** **spp. detection, identification and subtyping**

**Figure 1.**
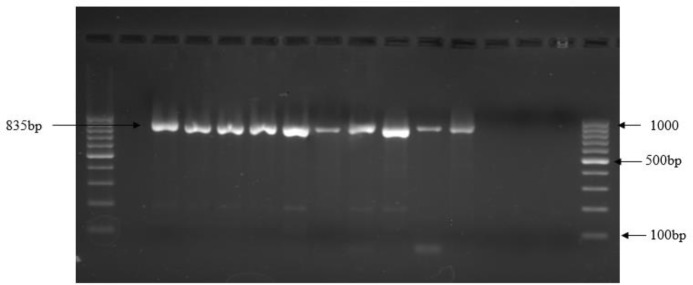
PCR assay for small subunit ribosomal (SSU rRNA) RNA gene of *Cryptosporidium* species

Oocysts of *Cryptosporidium* spp. measuring 4-6 µm by micrometry were detected upon microscopic examinations of modified acid-fast stained smears of stool samples. Only 41 cases were positive for oocysts of *Cryptosporidium* spp. by microscopy. Using SSU-rRNA PCR assay, two additional cases of cryptosporidiosis were detected making it to a total 43 cases of human cryptosporidiosis (Fig. 1; Table 2). 

**Table 1:** Distribution of *Cryptosporidium* spp. in adults and children according to underlying disease conditions

Underlying conditions	Total Number (n=307) Adults	*Cryptosporidium* Positive (n=26) (%)	Total number (n=143)Children	*Cryptosporidium* Positive (n=17) (%)
HIV seropositives	53	9 (17%)	15	6 (40%)
Malignant Diseases	9	1 (11%)	32	2 (6%)
Transplant Recipients	57	16 (28%)	5	2 (40%)
Primary immunodeficiency diseases	10	-	9	2 (22%)
Secondary immunodeficiency diseases	10	-	8	1 (13%)
Chronic disease	14	-	2	1 (50%)
No apparent immunocompromised condition	154	-	72	3 (42%)

**Table 2:** Distribution of subtypes within the subtype families

Subtype family	No. of Subtypes	Distribution of Subtypes
Ia	5	IaA17R2 (1) IaA20R2 (2) IaA23G1R1 (1) IaA29G2R2 (1)
Ib	6	IbA9G3 (5) IbA10G2 (1)
Id	7	IdA15G1 (5) IdA14 (2)
Ie	12	IeA11G3T3 (5) IeA11G3T1 (2) IeA13G3T3 (2) IeA13G3T1 (3)
IIa	2	IIaA15G3 (2)
IIc	3	IIc5G3 (3)
IId	3	IIdA15G1 (3)
IIe	1	IIeA7G1 (1)

**Figure 2(a and b).**
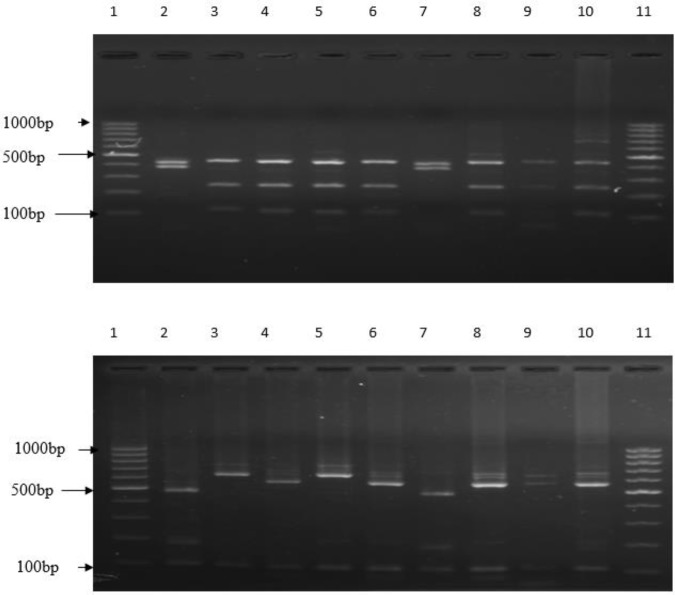
PCR-RFLP analysis using *Ssp*I and *AseI* restriction enzyme for differentiating *Cryptosporidium* species

Of these 43 positive samples, 70% (n=30; 30/43) were *C. hominis* and 21% (n=9; 9/43) were *C. parvum. *Only 7% (n=3; 3/43) were *C. felis* and 2% (n=1; 1/43) was *C. viatorum* (Fig. 2a and 2b). None of the stool samples from healthy controls were positive for *Cryptosporidium* spp. either by microscopy and/or PCR assay.

Sequence analysis of gp60 gene could be done for 30 *C. hominis* and 9 *C. parvum *and that could detect eight subtype families. These subtype families were Ia (13%, 5/39), Ib (15%, 6/39), Id (18%, 7/39), Ie (30%, 12/39), IIa (5%, 2/39), IIc (8%, 3/39), IId (8%, 3/39) and IIe (3%, 1/39). Amongst various subtypes, the frequency of occurrence of subtypes IbA9G3, IdA15G1 and IeA11G3T3 was relatively higher compared to others (Table-2).

**Figure 3(a and b).**
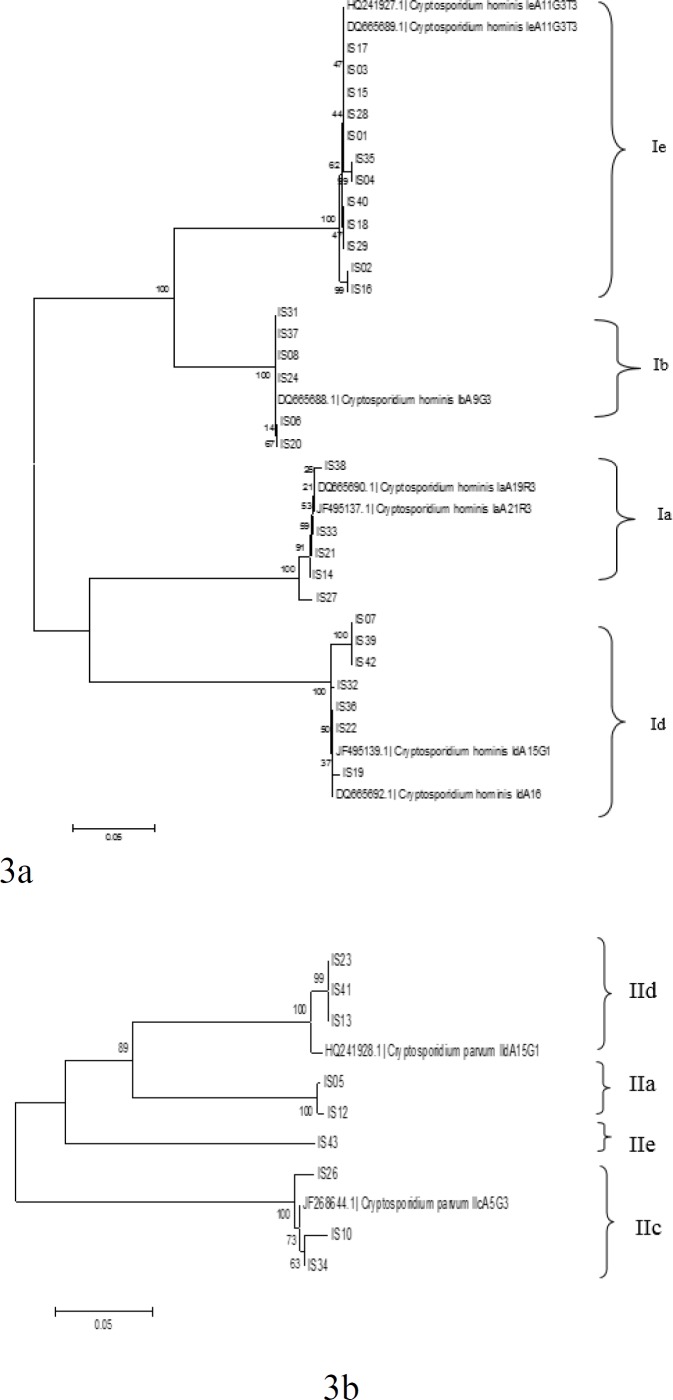
Phylogenetic relationship of *C. hominis* and *C. parvum* identified in the present study. Phylogenetic analysis inferred by neighbor-joining analysis of the gp60gene sequence based on evolutionary distances calculated using the Kimura two-parameter model. Bootstrap values were obtained using 1,000 pseudo replicates

*C. hominis* subtypes identified in the present study formed two different clades upon phylogenetic analysis (Fig. 3). *C. hominis* subtype family Ie and Ib were grouped together in one clade whereas subtype family Ia and Id were in another. Separation of these two-subtype family (Ie and Ib and Ia and Id) in two different clades was statistically reliable (bootstrap values 100%). Similarly, *C. parvum* also formed two clades with IIc subtypes in one and IIa, IId and IIe clustered in another. The discriminatory power (D) for gp60 subtyping was 0.83 in the present study. 

**Clinical manifestations**

When the clinical manifestations of infected individuals were correlated with different infecting subtypes, it was observed that all patients infected with subtype family Ia, Id and IIc had chronic diarrhea, whereas 83% (5/6) patients infected with Ib subtype family subtype family complained of vomiting and appetite loss. Patients infected with Ie subtype family showed higher frequency of passage of stools in a day. Specific clinical manifestation could not be ascertained in patients infected with other subtypes Clinical manifestations between the subtypes and their statistical association has been shown in Table 3.

## Discussion

The findings obtained in the present study showed that patients attending our tertiary care centre were infected with diverse population of *Cryptosporidium* species including *C. felis and C. viatorum*. *C. hominis* was the most common species (70%) responsible for cryptosporidiosis in our study followed by *C. parvum. *Similar reports of higher prevalence of *C. hominis* compared to other species have been reported in other developing countries including Brazil (24), Peru (10, 11), Kenya (25), Vietnam (26), Iran (27) and Haiti (28) and India (22, 29,30). 

**Table 3:** Clinical presentation in patients infected with different subtypes of *Cryptosporidium hominis* and *Cryptosporidium parvum*

Clinical manifestations	Ia (n=5)	Ib (n=6)	Id (n=7)	Ie (n=12)	IIa (n=2)	IIc (n=3)	IId (n=3)	IIe (n=1)	p-values
Chronic diarrhea	5 (100)^*^	3 (50)	7 (100)	7 (58)	1 (50)	3 (100)	-	-	0.09
Frequency more than 5	2 (40)	2 (33)	2 (29)	8 (66)	1 (50)	2 (67)	-	1 (100)	0.5
Blood in stools	1 (20)	1 (17)	-	-	-	-	-	-	0.88
Mucus in stools	3 (60)	1 (17)	2 (29)	3 (25)	-	-	2 (67)	-	0.36
Abdominal pain	2 (40)	1 (17)	3 (43)	6 (50)	-	2 (67)	-	1 (100)	0.56
Bloating	2 (40)	2 (33)	4 (57)	4 (33)	-	2 (67)	-	1 (100)	0.66
Fever	2 (40)	3 (50)	4 (57)	5 (42)	-	2 (67)	1 (33)	1 (100)	0.88
Vomiting	1 (20)	5 (83)	2 (29)	8 (66)	-	1 (33)	1 (33)	1 (100)	0.14
Appetite loss	2 (40)	5 (83)	2 (29)	2 (17)	-	2 (67)	1 (33)	1 (100)	0.11
Weight loss	4 (80)	1 (17)	3 (43)	5 (42)	-	1 (33)		1 (100)	0.31
Ulcers	-	1 (17)	2 (29)	1 (8.3)	-	2 (67)	-	1 (100)	0.09

* No. (%)

Differences in geographical distribution of *C. hominis* and *C. parvum* are generally considered as a reflection of differences in both sources of infection and routes of transmission (5). Transmission of *C. hominis* is essentially anthroponotic and that of *C. parvum* excluding the subtype families IIc and IIe are primarily zoonotic. The predominance of *C. hominis* in most of the developing countries suggests that anthroponotic transmission is more common and important than zoonotic transmission in the epidemiology of human cryptosporidiosis (5). Certain subtype such as “If” could not be identified in the present study although such subtype has been reported from one of the study from India (29). The distribution of *C. parvum *subtypes in this study suggests the likely occurrence of zoonotic transmission at a much lower frequency. *C. parvum* subtype family IId was identified in only three (n=3) cases. Subtype family IId of *C. parvum* is more commonly found in sheep and goats (31), but has also been reported from amongst calves in China, Egypt, and Sweden (32-34). 

The existence of many subtypes within *C. hominis* and *C. parvum* subgenotype families in the present study reinforces the complexity of *Cryptosporidium* spp. transmission, and its association with different clinical presentation (5). Many subtype alleles were observed in the phylogenetic analysis for *C. hominis* subtype families. The high heterogeneity of* C. hominis* shown in the phylogenetic tree is thought to express intensive and stable anthroponotic transmission of cryptosporidiosis in the present study. Similarly, recent study from Nigeria showed high heterogeneity of *C. hominis* compared to *C. parvum* (35). The discriminatory power for gp60 subtyping is high in the study setting (D = 0.83) making it a suitable tool for the detection of outbreaks in human-to-human transmission settings (8). Amongst* C. parvum *species, only one subtype allele was observed from all four subtypes identified in the present study. From earlier studies conducted in India one subtype allele of IIc (IIcA5G3) and IIe(IIeA7G1) as well as two alleles of IId have been reported (16,23).

Variations in the clinical presentations were observed among *C*. *hominis *and* C. parvum *subtype families (Table -3). Various studies have shown the association of subtype family with diarrhea (10,11), abdominal pain (34), fever and dehydration (36). In the present study, we could observe association of Ia and Id subtype with chronic diarrhea. Cama et al., had reported the association of Ia with diarrhea and Ib with vomiting, however the results were not statistically significant. Infection with Ib subtype family has been reported to be much more virulent than other subtype families (10,11). Recent study from Ethiopia showed significant association of *C. parvum* subtype family IIa with diarrhea (37)

Present study reported four *Cryptosporidium* species and 16 subtypes belonging to 8 subtype families of *C. hominis* and *C. parvum* in Indian patients. The study results provided updated molecular evidence on the diversity and frequency of the *C. hominis *and *C. parvum* subtypes currently circulating in symptomatic individuals seeking medical care at our tertiary care centre. The results may have further implications in better understanding of the changing epidemiology and transmission dynamics of cryptosporidiosis.
